# BREF: the value of a short early intervention for informal carers and families for reducing stress and burden of care

**DOI:** 10.1192/j.eurpsy.2025.91

**Published:** 2025-08-26

**Authors:** E. Scanferla, R. Rey

**Affiliations:** 1 CRPMS (EP), Université de Paris; 2 GHU Paris psychiatrie et neurosciences, Paris; 3 Centre Lyonnais des Aidants en Psychiatrie (CLAP), Le Vinatier-Psychiatrie Universitaire Lyon Métropole, Bron; 4 Fondation FondaMental, Créteil; 5 U1028/UMR5292, INSERM/CNRS, Lyon, France

## Abstract

**Abstract: Introduction:**

Informal caregivers are essential in supporting individuals facing mental health challenges, yet the demanding nature of their role can lead to significant distress and long-term psychological strain. International guidelines consistently highlight the critical importance of early caregiver support and identify psychoeducation for caregivers as one of the most effective interventions to support them. However, psychoeducational programmes for caregivers remain significantly underutilised. To address this pressing gap in mental health support, Rey et al. (2020), in collaboration with Unafam, the French national family association, developed the BREF programme. This innovative psychoeducational intervention is designed to provide early and systematic support for caregivers of people with severe mental disorders.

**Objectives:**

This study investigated the impact of the BREF programme, focusing on its potential to mitigate depressive symptoms and alleviate the psychological burden experienced by caregivers.

**Methods:**

This study used a single-group pre-post design. It included family caregivers who participated in the BREF programme from November 2020 to March 2022. Changes in caregiver depressive symptoms (CES-D) and burden (ZBI) measured pre-, post- and 3 months after intervention. Caregivers’ satisfaction and perceived usefulness were also assessed.

**Results:**

Data from 206 family caregivers were analysed. The depression and burden scores significantly decreased immediately after the intervention (p<0.001) and at the 3-month follow-up (p<0.05). Additionally, 98% of participants reported being satisfied to very satisfied, 95% of them deemed it very to extremely useful.

**Conclusions:**

The BREF programme demonstrated significant benefits, notably reducing caregivers’ depressive symptoms and burden. Designed for early systematic implementation this standardized, time- and resource- efficient intervention, offers à promising foundation for a structured and graduated support pathway for caregivers.

**Disclosure of Interest:**

None Declared

